# Draft Genome Sequences of 11 *Salmonella enterica* Serovar Typhimurium Strains Isolated from Human Systemic and Nonsystemic Sites in Brazil

**DOI:** 10.1128/genomeA.01223-17

**Published:** 2018-02-01

**Authors:** Pedro Henrique N. Panzenhagen, Narayan C. Paul, Carlos A. Conte Junior, Renata G. Costa, Dália P. Rodrigues, Devendra H. Shah

**Affiliations:** a Department of Veterinary Microbiology and Pathology, Washington State University, Pullman, Washington, USA; b Food Science Program, Chemistry Institute, University Federal of Rio de Janeiro, Rio de Janeiro, Brazil; c National Reference Laboratory for Diagnosis of Enteric Bacteria, Oswaldo Cruz Institute, Rio de Janeiro, Brazil; d National Institute of Quality Control in Health, Oswaldo Cruz Foundation, Rio de Janeiro, Brazil; e Department of Food Technology, Faculty of Veterinary Medicine, Federal Fluminense University (UFF), Niterói, Brazil

## Abstract

*Salmonella enterica* serovar Typhimurium strains isolated from systemic sites outside sub-Saharan Africa have been rarely sequenced. Here, we report the draft genome sequences of *S*. Typhimurium sequence type 19 (ST19) (*n* = 9), ST1649 (*n* = 1), and ST313 (*n* = 1) strains isolated from human systemic (e.g., blood) and nonsystemic (e.g., stool and wounds) sites in Brazil.

## GENOME ANNOUNCEMENT

Nontyphoidal *Salmonella* (NTS) is one of the major causes of diarrheal disease worldwide, with an estimated 93 million enteric infections and 155,000 deaths annually (1). However, these estimates do not include the infections caused by invasive nontyphoidal *Salmonella* (iNTS) *enterica* serovar Typhimurium sequence type 313 (ST313), which is often associated with systemic infection. *S*. Typhimurium ST313 strains account for >50% of the systemic infections in sub-Saharan Africa (2, 3). Two genetic lineages of *S*. Typhimurium ST313 have been linked with the emergence of iNTS across sub-Saharan Africa (4). In Brazil, *S*. Typhimurium is frequently isolated from systemic sites, such as blood and cerebrospinal fluid (CSF), from human patients (5, 6); however, the genetic underpinning of *S*. Typhimurium isolates recovered from systemic sites in Brazil remains unknown. We sequenced 11 *S*. Typhimurium strains isolated from blood (*n* = 4), stool (*n* = 4), and extraintestinal (*n* = 3) sites between 2010 and 2014 from different geographic regions of Brazil (Table 1).

**TABLE 1:** GenBank accession numbers of *Salmonella* Typhimurium strains isolated from human clinical samples in Brazil

Strain	SRA accession no.	WGS accession no.^a^	No. of contigs	*N*_50 _(bp)	Length (bp)	Source^b^	Yr	State
PP_BR007	SAMN05505504	NPJP00000000	156	57,229	4,632,574	Blood	2014	Rio de Janeiro
PP_BR026	SAMN05505503	NPJQ00000000	149	62,099	4,777,031	Blood	2012	Rio Grande do Sul
PP_BR027	SAMN05505502	NPJR00000000	175	44,398	4,626,743	WS	2012	Rio Grande do Sul
PP_BR031	SAMN05505501	NPJS00000000	190	46,721	4,594,320	WS	2012	Rio Grande do Sul
PP_BR032	SAMN05505500	NPJT00000000	122	99,313	4,650,556	ABS	2012	Rio Grande do Sul
PP_BR045	SAMN05505499	NPJU00000000	147	76,702	4,827,606	RS	2011	Santa Catarina
PP_BR057	SAMN05505498	NRDL00000000	131	103,073	4,787,663	Blood	2011	Amapá
PP_BR060	SAMN05505496	NPMT00000000	123	96,334	4,776,943	Feces	2011	Minas Gerais
PP_BR062	SAMN05505495	NRDM00000000	118	142,310	4,958,705	Feces	2010	Santa Catarina
PP_BR063	SAMN05505494	NRDN00000000	161	50,016	4,365,189	Blood	2010	Rio Grande do Sul
PP_BR076	SAMN05505493	NSDQ00000000	125	120,490	5,065,902	Feces	2010	Rio Grande do Sul

a WGS, whole-genome sequencing.

b WS, wound secretion; ABS, abdominal abscess; RS, rectal swab.

Paired-end sequencing libraries (2 × 250 bp) were prepared using the Nextera XT kit (Illumina, San Diego, CA) following the protocol described in the DNA library reference guide (7), size selected to be in the range of 600 to 1,000 bp (average peaks, ~800 bp), and sequenced using the MiSeq Illumina version 2 kit, according to the manufacturer’s instructions. The number of paired reads per sample ranged from 941,320 to 1,389,798. The average G+C content was 52.5% (8). *De novo* assembly was performed using Velvet 1.2.10 set at default for all parameters (9). Contigs were organized by aligning to the genome sequence of the reference strain *S*. Typhimurium LT2 (accession no. NC_003197) using Mauve multiple alignments (10). Whole-genome multilocus sequence typing was performed using EnteroBase (https://enterobase.warwick.ac.uk/). A total of three sequence types, ST19 (*n* = 9), ST1695 (*n* = 1), and ST313 (*n* = 1), were identified. Detailed comparative genomics analysis of these strains is currently ongoing and will be published independently. These genome sequences will provide better insights into the molecular epidemiology of invasive *S*. Typhimurium strains in Brazil.

### Accession number(s).

The draft genome sequences are available in GenBank under the accession numbers listed in Table 1.
